# Health outcomes and their association with weight regain after substantial weight loss in Sweden: a prospective cohort study

**DOI:** 10.1016/j.lanepe.2025.101261

**Published:** 2025-03-13

**Authors:** Lena M.S. Carlsson, Ida Arnetorp, Johanna C. Andersson-Assarsson, Peter Jacobson, Per-Arne Svensson, Magdalena Taube, Sofie Ahlin, Felipe M. Kristensson, Kristjan Karason, Ingrid Larsson, Cecilia Karlsson, Kirsi H. Pietiläinen, Ingmar Näslund, Björn Carlsson, Markku Peltonen, Kajsa Sjöholm

**Affiliations:** a Institute of Medicine, Sahlgrenska Academy at the University of Gothenburg, Gothenburg, Sweden; b Institute of Health and Care Sciences, Sahlgrenska Academy at the University of Gothenburg, Gothenburg, Sweden; c Region of Västra Götaland, NU Hospital Group, Department of Clinical Physiology, Trollhättan, Sweden; d Region Västra Götaland, Sahlgrenska University Hospital/Östra, Department of Surgery, Gothenburg, Sweden; e Departments of Cardiology and Transplantation, Sahlgrenska University Hospital, Gothenburg, Sweden; f Late-Stage Development, Cardiovascular, Renal and Metabolism (CVRM), BioPharmaceuticals R&D, AstraZeneca, Gothenburg, Sweden; g Obesity Research Unit, Research Program for Clinical and Molecular Metabolism, Faculty of Medicine, University of Helsinki, Helsinki, Finland; h Healthy Weight Hub, Abdominal Centre, Helsinki University Hospital and University of Helsinki, Helsinki, Finland; i Faculty of Medicine and Health, Department of Surgery, Örebro University, Örebro, Sweden; j Research and Early Development, Cardiovascular, Renal and Metabolism (CVRM), BioPharmaceuticals R&D, AstraZeneca, Gothenburg, Sweden; k Finnish Institute for Health and Welfare, Helsinki, Finland

**Keywords:** Obesity, Bariatric surgery, Weight regain, Morbidity, Mortality

## Abstract

**Background:**

The clinical implications of weight regain following weight loss remain uncertain. We analysed mortality, cardiovascular events, cancer, and microvascular disease in individuals with significant weight loss, comparing maintainers to regainers.

**Methods:**

Using a prospective cohort design, we analysed 1346 participants who underwent bariatric surgery in the Swedish Obese Subjects (SOS) study, aged 37–60 years with BMI ≥34 (men) or ≥38 (women), recruited 1987–2001. Individuals who regained ≥20% of their 1-year weight loss after 4 years (regain group) were compared to those who regained less (maintenance group). The study was closed on December 31, 2020 with median follow-up of 27 years and 99.9% mortality tracking (ClinicalTrials.govNCT01479452).

**Findings:**

Average weight loss after 1 year was 29.3 ± 11.7 kg and 31.9 ± 13.8 kg and average weight change from year 1 to year 4 was +12.7 ± 6.6 kg and −0.6 ± 7.3 kg in the regain and maintenance groups, respectively. During follow-up, regain and maintenance groups showed similar rates of total mortality and cancer, 12.4 (95% CI: 10.9–14.2) vs 12.4 (10.7–14.3), p = 0.740, and 11.3 (95% CI: 9.7–13.0) vs 10.4 (8.8–12.2) per 1000 person-years (p = 0.308), respectively. The regain group had, however, higher incidence of microvascular disease, 11.0 (95% CI: 9.5–12.8) vs 8.7 (7.3–10.4) per 1000 person-years (p = 0.024), and while not statistically significant, also higher incidence of major adverse cardiovascular events (myocardial infarction, stroke, and heart failure) 15.7 (95% CI: 13.8–17.8) vs 13.0 (11.2–15.1) per 1000 person-years (p = 0.055).

**Interpretation:**

Weight regain was linked to increased vascular disease risk but we could not demonstrate an association with life expectancy.

**Funding:**

The Swedish Research Council, the Swedish State under the agreement between the Swedish Government and the county councils, the Health & Medical Care Committee of the Region Västra Götaland, the Adlerbert Research Foundation, the Wilhelm and Martina Lundgren Foundation, the Royal Society of Arts and Sciences in Gothenburg, Academy of Finland, Finnish Medical Foundation, Gyllenberg Foundation, Novo Nordisk Foundation, Finnish Diabetes Research Foundation, Paulo Foundation and Sigrid Juselius Foundation.

**Research in context**

**Evidence before this study**

Weight regain following weight loss is very common and remains a significant challenge in obesity management. For example, potent weight loss drugs effectively reduce body weight, but rapid weight regain often occurs after discontinuation or dosage reduction. Similarly, weight loss from lifestyle interventions is often temporary, with weight frequently regained. Bariatric surgery usually leads to rapid weight loss, peaking within one to two years, but partial weight regain is common, as shown by many studies. We searched PubMed from inception to September 18, 2024, using the terms “weight regain” “weight loss” “intervention” and “mortality” and found no studies.

**Added value of this study**

To our knowledge, this is the first study to investigate whether long-term morbidity and mortality following significant weight loss differ based on whether the initial weight loss is maintained or partially regained. We examined health outcomes over 30 years after bariatric surgery, comparing individuals who partly regained their initial weight loss with those who maintained their weight loss. Those who regained weight exhibited a less favourable cardiovascular risk profile and experienced higher rates of both microvascular and macrovascular events. However, their mortality rate was similar to that of those who maintained their weight loss. Importantly, both groups had lower mortality rates than the control group receiving standard obesity care.

**Implications of all the available evidence**

Partial weight regain after substantial weight loss was linked to a higher risk of microvascular and macrovascular diseases, but no association with life expectancy was identified.

## Introduction

Obesity increases the risk of numerous health problems, leading to a reduction in life expectancy.^1^^,^^2^ Weight regain following weight loss is very common and remains a significant challenge in obesity management.^3^ For example, treatment with semaglutide effectively reduces body weight, but once the medication is discontinued or the dosage is reduced, rapid weight regain often occurs.^4^ Similarly, weight loss through lifestyle interventions is often short-lived, with the weight frequently regained later on.^5^ Bariatric surgery typically results in rapid weight loss, reaching its peak within one to two years, but partial weight regain is a common issue, as shown by numerous studies.^6^, ^7^, ^8^, ^9^, ^10^

Until recently, bariatric surgery was the only treatment that achieved significant, long-lasting weight loss, and it remains the only obesity treatment that has been associated with substantial reductions in the risk of developing type 2 diabetes,^11^ macrovascular^12^^,^^13^ and microvascular diseases,^13^^,^^14^ cancer,^15^^,^^16^ and an increased life expectancy.^10^^,^^17^ An incentive for individuals opting for bariatric surgery is to mitigate the risks of declining health and premature mortality.^18^ Patients perceive weight regain as a difficult experience, fearing its impact on their future health.^19^ Given the long-lasting improvement of health outcomes following bariatric surgery, it appears that weight-loss maintenance is an essential mechanism, but substantial evidence supporting this notion remains sparse. To the best of our knowledge, no study has specifically investigated whether long-term health outcomes following significant weight loss vary depending on whether the initial weight loss is maintained or followed by partial weight regain. The aim of this study was to examine the relationship between weight regain after substantial weight loss and the incidence of cardiovascular disease, cancer, and microvascular disease, as well as cause-specific mortality and life expectancy over a 30-year period in the prospective Swedish Obese Subjects (SOS) study.

## Methods

### Study design

The prospective matched SOS study, designed to compare overall mortality between individuals treated with bariatric surgery and usual obesity care, has been previously described.^10^^,^^20^ In brief, 4047 individuals were enrolled between 1 September 1987 and 31 January 2001. A bariatric surgery group of 2007 individuals was formed along with a matched control group of 2040 participants (Supplementary Figure S1). The study was conducted at 25 public surgical departments and 480 primary health care centres in Sweden. Seven regional ethics review boards (Gothenburg, Lund, Linköping, Örebro, Karolinska Institute, Uppsala, Umeå) approved the study protocol, and written or oral informed consent was obtained from all participants. This consent procedure was reviewed and approved by all seven review boards, approval number 184-90, date of decision June 6, 1990 (renewed approval number T508-17, date of decision June 12, 2017). The study has been registered at ClinicalTrials.gov (NCT01479452).

The inclusion criteria were ages 37–60 years and a body mass index (BMI) of at least 34 in men and 38 or more in women. The exclusion criteria were previous surgeries for gastric or duodenal ulcers, prior bariatric surgery, gastric ulcer in the past 6 months, ongoing or active malignancy within the past 5 years, myocardial infarction in the past 6 months, bulimic eating patterns, drug or alcohol abuse, psychiatric or cooperative problems contraindicating bariatric surgery, or other contraindicating conditions. Baseline examinations took place 4 weeks before the intervention. Follow-up visits, including physical examinations and questionnaires, were scheduled at 0.5, 1, 2, 3, 4, 6, 8, 10, 15, and 20 years. Fasting blood samples were collected at baseline and after 2, 10, 15, and 20 years.

The surgery group underwent banding (18%), vertical banded gastroplasty (69%) or gastric bypass (13%). Participants who had a reoperation with a different surgical technique (n = 372, 18.5%), or an operation which reinstated normal anatomy (n = 102, 5.1%), were excluded. The control group received conventional obesity treatment.

The surgery group was divided into weight regain and maintenance groups based on weight changes between baseline and the 1-year follow-up, and between 1- and 4-year follow-up examinations. Those who regained 20% or more of their 1-year weight loss by the 4-year examination were assigned to the weight regain group, while those who regained less than 20% formed the weight maintenance group.^6^ In a sensitivity analysis, a threshold of 30% weight regain^21^ was used to further validate the findings. Participants were required to have body weight data at baseline, as well as at the 1- and 4-year follow-up to allow for assessment of initial weight loss and subsequent weight regain. Consequently, for the remaining individuals, those with missing data on body weight (n = 172, 11.2%) or who died (n = 15, 1.0%) within the first four years were excluded (Supplementary Figure S1).

Mortality data until December 31, 2020, and official causes of death, were obtained from the Swedish Population and Address Register and the Swedish Cause of Death Register, respectively. Relevant case sheets and autopsy reports were independently evaluated by two authors. If there was a discrepancy between the study-determined and official cause of death, the study-determined direct cause was used, as the official cause often reflects an underlying preventable cause like obesity.^22^

Fatal and non-fatal major adverse cardiovascular events (myocardial infarction, stroke, and heart failure) and microvascular disease (retinopathy, diabetic kidney disease, and neuropathy) were identified from the Swedish Cause of Death Register and the Swedish National Patient Register, respectively, using International Classification of Diseases codes and intervention codes (Supplementary Table S1). This register includes diagnoses for inpatients since 1987 and hospital-based outpatient visits since 2001. Reporting is mandatory for public hospitals and coverage is 99%.^23^ Cancer incidence data were obtained from the Swedish National Cancer Registry, covering more than 95% of all malignant tumors.^24^ Type 2 diabetes was defined by a fasting blood glucose level ≥6.1 mmol/L, HbA1c ≥ 48 mmol/mol, and/or self-reported use of anti-diabetes medication.

### Statistical analyses

Demographics of the participants are presented as mean values with standard deviations or as frequencies and percentages. Baseline comparisons between the groups used analysis of covariance for continuous variables and a Fisher's exact test for dichotomous variables.

The differences in the changes in BMI and risk factors between the groups were analysed with multilevel mixed-effects regression models. The observations were considered nested within the participants. For individuals who dropped out of the study, we assumed missing data were “missing at random” (i.e. missing data are only dependent on the participant's observed data), and all the observed data were used in the analyses.

For time-to-event analyses, participants were followed until first event, death, emigration, or end of register-based follow-up (December 31st, 2020), whichever came first. Participants who emigrated, died, or did not experience an event during follow-up were treated as censored observations at the corresponding date.

Kaplan–Meier estimates and mortality rates were calculated for each group. Statistical inference was done with Cox and the Gompertz proportional hazard regression models and the results are presented as hazard ratios (HR) and differences in median life-expectancy with corresponding confidence intervals (CIs). The proportional hazard assumption was evaluated by assessing the interaction between weight maintenance group and logarithm of time, and with inspection of log-log plots. In cases where the assumption was not fulfilled, the Cox models were modified to allow time-varying coefficients, and in these cases the results are presented as time-interval specific HRs.

Adjusted analyses considered preselected predictors (age, sex, BMI, smoking status, diabetes, history of cardiovascular disease, year of inclusion to the study, and the type of surgery). Analyses on cause-specific mortality were conducted with competing-risks regression models suggested by Fine and Gray, in which deaths for other reasons were treated as competing events.^25^

The cut-off date for register linkages was December 31, 2020. Data from participants who emigrated (n = 19), withdrew consent (n = 3), or were alive at the end of follow-up (December 31, 2020; n = 946) were censored on the corresponding date in the time-to-event models.

All statistical tests were two-tailed and p values of less than 0.05 were considered statistically significant. Stata statistical package 15.1 (StataCorp, 2017. Stata Statistical Software: Release 15. College Station, TX: StataCorp LLC) was used.

### Role of the funding source

The funders of the study had no involvement in the design, data collection, analysis, interpretation, or preparation of the manuscript. All authors had full access to the data and approved the submission of the manuscript for publication.

## Results

### Participants

After exclusion of individuals with reoperations, missing data, or who died within the first four years, 1346 (67.1%) individuals were available for assignment to weight maintenance or weight regain groups (Supplementary Figure S1). The baseline characteristics of weight regain (n = 715) and weight maintenance groups (n = 631), are shown in Table 1. In both groups the majority of participants were women (70%). Baseline BMI was lower in the weight regain group compared to the weight maintenance group (41.9 ± 4.1 kg/m^2^ vs 42.8 ± 4.5 kg/m^2^). Other characteristics were similar in both groups. Median follow-up was 27 years (interquartile range 24–29). Weight regain was influenced by the type of surgery, with gastric bypass resulting in fewer relapses than vertical banded gastroplasty.

**Table 1:** Baseline characteristics.

Variable	Regain (n = 715)^a^	No. with missing data	Maintenance (n = 631)^a^	No. with missing data
Age — yr	47.9 ± 6.0	0	47.7 ± 6.1	0
Male Sex — n (%)	215 (30.1)	0	192 (30.4)	0
Body Mass Index^b^	41.9 ± 4.1	0	42.8 ± 4.5	0
Waist-to-hip ratio	0.988 ± 0.079	3	0.992 ± 0.079	0
Cardiovascular disease before baseline — n (%)	18 (2.5)	0	13 (2.1)	0
Cancer before baseline — n (%)	9 (1.3)	0	12 (1.9)	0
Hypertension — n (%)	586 (82.1)	1	501 (79.4)	0
Glucose status — n (%)^c^				
Normal	371 (52.0)	2	327 (52.1)	3
Impaired	198 (27.8)	2	176 (28.0)	3
Type 2 diabetes	144 (20.2)	2	125 (19.9)	3
Insulin level — mU/liter	21.3 ± 14.9	3	21.1 ± 11.8	4
Daily smoking — n (%)	143 (20.0)	0	148 (23.5)	1
Total cholesterol — mmol/liter	5.9 ± 1.2	3	5.9 ± 1.1	1
Physically active — n (%)				
Leisure time physical activity	402 (56.4)	2	339 (53.8)	1
Work-related physical activity	442 (61.9)	1	359 (57.1)	2
Energy intake — kcal/day	2808 ± 1184	0	2909 ± 1151	0
University education — n (%)	92 (12.9)	0	70 (11.1)	0
Married or living with partner — n (%)	537 (75.2)	1	460 (73.0)	1
Type of surgery — n (%)				
Gastric bypass	77 (10.8)	0	153 (24.2)	0
Gastric banding	104 (14.5)	0	119 (18.9)	0
Vertical banded gastroplasty	534 (74.7)	0	359 (56.9)	0

a Plus-minus values are means ± SD. There were imbalances (p < 0.05) between the regain and maintenance groups with respect to baseline body mass index and type of surgery.

b The body mass index is the weight in kilograms divided by the square of the height in meters.

c Glucose status was classified as normal: fasting blood glucose level <5.0 mmol/L (<90 mg/dL) and HbA1c <39 mmol/mol; impaired: ≥5.0 to < 6.0 mmol/L (≥90–<110 mg/dL) or HbA1c 39–47 mmol/mol; type 2 diabetes: HbA1c ≥48 mmol/mol or fasting blood glucose of ≥6.1 mmol/L (≥110 mg/dL), or diabetes medication use.

### BMI, energy intake, and physical activity

**Fig. 1:**
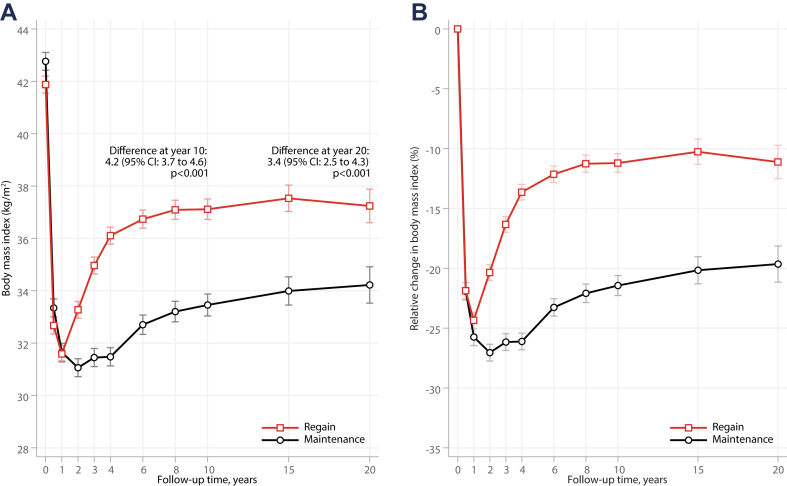
**Changes in body mass index, both absolute (A) and relative (B), over 20 years following bariatric surgery for the weight regain and weight maintenance groups.** The comparison between the regain and the maintenance group was adjusted for age and sex.

**Fig. 2:**
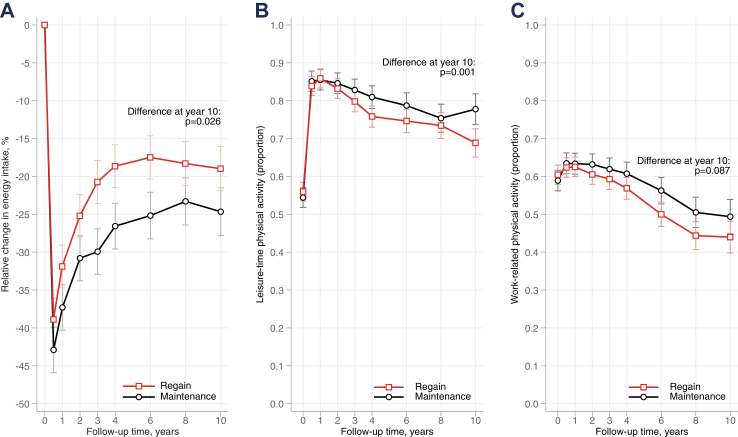
**Self-reported energy intake (A), leisure-time physical activity (B), and work-related physical activity (C) in the weight regain and weight maintenance groups over 10 years following bariatric surgery.** The comparisons between the regain and the maintenance group were adjusted for age and sex.

Changes in BMI over 20 years for the weight regain and maintenance groups are depicted in Fig. 1. At the 1-year examination, the regain and maintenance groups had lost 29.3 ± 11.7 and 31.9 ± 13.8 kg, respectively. From year 1 to year 4, average weight change was +12.7 ± 6.6 and −0.6 ± 7.3 kg in the regain and maintenance groups, respectively. The difference in BMI between the groups observed at year 4 largely persisted over 20 years. Waist circumference showed a similar pattern, with both groups experiencing a rapid decrease in the first year, followed by more pronounced increase in the regain group (Supplementary Figure S2). At the 10-year follow-up, the weight regain group reported higher energy intake and lower leisure-time physical activity levels than the maintenance group, as shown in Fig. 2. The number of individuals with available data at different time-points for Fig. 1, Fig. 2 are shown in Supplementary Table S2.

### Changes in cardiovascular risk factors

Two years after bariatric surgery, improvements were observed across all measured indicators of glucose and lipid metabolism, alongside reductions in both systolic and diastolic blood pressure, for participants in both the weight regain and weight maintenance groups (Supplementary Figures S3–S5). Between year two and year 10, glucose metabolism markers had deteriorated in both groups, but were significantly worse in the regain group (Supplementary Figure S3). Furthermore, individuals in the regain group, free from type 2 diabetes at baseline, had higher incidence of type 2 diabetes (HR = 2.01, 95% CI: 1.40–2.89, p < 0.001), compared to the maintenance group. Total and LDL cholesterol levels were similar in the groups at year 10, while HDL cholesterol and triglycerides were worse in the regain group (Supplementary Figure S4). By year 10, both groups experienced increased blood pressure, with the regain group showing higher systolic levels (Supplementary Figure S5).

### Incidence of major adverse cardiovascular events, cancer and microvascular disease

**Fig. 3:**
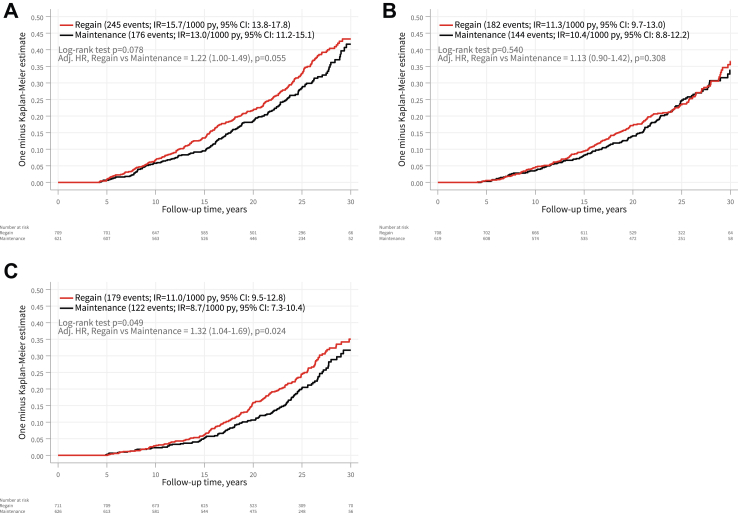
**Kaplan–Meier cumulative incidence of major adverse cardiovascular events (myocardial infarction, stroke and heart failure) (A), cancer (B), and microvascular disease (C) in the weight regain and maintenance groups after bariatric surgery, including both fatal and non-fatal events.** The comparisons between the regain and the maintenance group were adjusted for age, sex, BMI, smoking status, diabetes, history of cardiovascular disease, year of inclusion to the study, and the type of surgery. Individuals with an event during the first four years were excluded from the respective outcome analysis.

During follow-up, there were 245 major adverse cardiovascular events (myocardial infarction, stroke, and heart failure) in the regain group and 176 in the maintenance group (Fig. 3). The event rates were 15.7 per 1000 person-years (95% CI: 13.8–17.8) for the regain group and 13.0 per 1000 person-years (95% CI: 11.2–15.1) for the maintenance group. The adjusted hazard ratio (HR) was 1.22 (95% CI: 1.00–1.49), with a borderline p value of 0.055. In a sensitivity analysis, where individuals who regained 30% or more of their one-year post-surgery weight loss by the 4-year examination were classified as part of the regain group, the incidence of major adverse cardiovascular events was significantly higher in the regain group (adjusted HR 1.35 [95% CI: 1.11–1.65], p = 0.003) (Supplementary Figure S6). The incidence of cancer was comparable between the two groups, with 182 events in the regain group and 144 events in the maintenance group. The corresponding rates were 11.3 per 1000 person-years (95% CI: 9.7–13.0) for the regain group and 10.4 per 1000 person-years (95% CI: 8.8–12.2) for the maintenance group (p = 0.308). Microvascular disease was more prevalent in the regain group, with 179 events, compared to 122 events in the maintenance group. This resulted in incidence rates of 11.0 per 1000 person-years (95% CI: 9.5–12.8) for the regain group and 8.7 per 1000 person-years (95% CI: 7.3–10.4) for the maintenance group, with an adjusted HR of 1.32 (95% CI: 1.04–1.69, p = 0.024). The sensitivity analysis, using a threshold of 30% weight regain, confirmed the findings for both cancer and microvascular disease (Supplementary Figure S6).

### Overall mortality and life expectancy

**Fig. 4:**
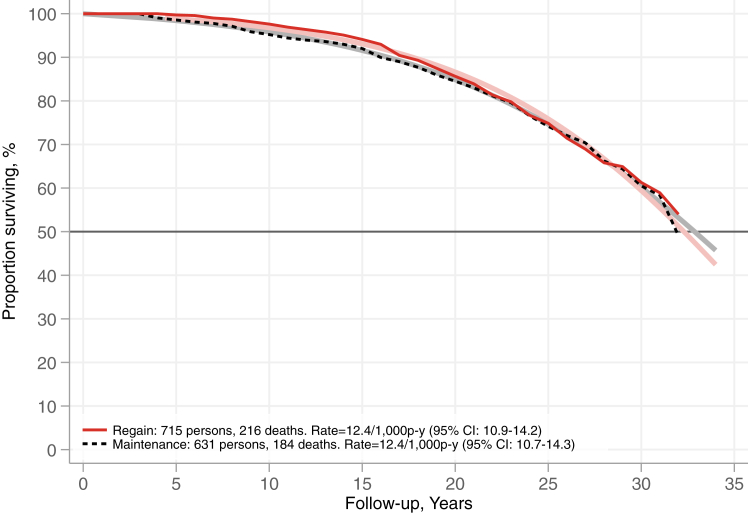
**Survival in weight regain and maintenance groups.** Shown are the Kaplan–Meier estimate of survival (opaque lines) and the estimate of survival from an unadjusted Gompertz regression model (fainter lines).

During follow-up, there were 216 deaths in the regain group and 184 in the maintenance group, resulting in nearly identical overall mortality rates of 12.4 per 1000 person-years for both groups (95% CI for regain: 10.9–14.2, maintenance: 10.7–14.3), p = 0.740, as shown in Supplementary Figure S7. The assumption of proportional hazards was not fulfilled for total mortality (test of time and weight maintenance group interaction, adjusted p = 0.032). Initially, the regain group exhibited lower mortality rates, but these rates became more similar in later phases of follow-up, with adjusted HRs for the regain vs maintenance group of 0.54 (95% CI: 0.30–0.97) at 5 years and 1.04 (95% CI: 0.85–1.28) at 20 years. Median life expectancy was similar between the groups, with the regain group having an estimated life expectancy 0.6 years shorter (95% CI: −2.7 to 1.6) than the maintenance group (Fig. 4). Both the regain and the maintenance group had lower mortality rate compared to the SOS control group receiving usual obesity care (mortality rate ratio 0.80 [95% CI: 0.68–0.94] and 0.83 [95% CI: 0.69–0.99] vs control group, respectively) (Supplementary Figure S7).

In a sensitivity analysis that assigned individuals who regained 30% or more of their one-year post-surgery weight loss by the 4-year examination to the regain group, similar mortality rates in the regain and maintenance groups were confirmed (Supplementary Figure S8). Moreover, stratified analyses by surgery type also showed comparable mortality rates between the groups (Supplementary Figure S9).

### Cause-specific mortality

Cardiovascular disease and cancer were leading causes of death in both groups, as shown in Supplementary Figure S10. The number of deaths from cardiovascular disease was 77 in the regain group and 57 in the maintenance group, corresponding to similar incidence rates of 4.4 per 1000 person-years (95% CI: 3.5–5.5) and 3.8 per 1000 person-years (95% CI: 3.0–5.0), respectively (p = 0.550). Cancer mortality was also comparable, with 64 vs 62 deaths, resulting in incidence rates of 3.7 per 1000 person-years (95% CI: 2.9–4.7) for the regain group and 4.2 per 1000 person-years (95% CI: 3.3–5.4) for the maintenance group, respectively (p = 0.399). Deaths from other causes followed a similar pattern, with 75 vs 65 deaths, resulting in incidence rates of 4.3 per 1000 person-years (95% CI: 3.4–5.4) for the regain group and 4.4 per 1000 person-years (95% CI: 3.4–5.6) for the maintenance group, respectively (p = 0.849).

## Discussion

In this study, we examined health outcomes over a 30-year period, comparing individuals who regained weight after an initial weight loss with those who maintained their weight loss. Although individuals who regained weight exhibited a less favourable cardiovascular risk profile and experienced higher incidences of both microvascular and macrovascular events, their mortality rate remained comparable to those who maintained their weight loss. Additionally, both the regain and maintenance groups had lower mortality rates compared to the SOS control group receiving standard obesity care. These findings suggest that even after partial weight regain, the positive association between bariatric surgery and increased life expectancy persists.

The definitions used to identify weight regain after bariatric surgery vary significantly across studies, substantially affecting the proportion of individuals classified as experiencing weight regain.^7^^,^^26^ A prospective study, aiming for standardized measurement, found that weight regain, expressed as a percentage of maximal weight loss, exhibited the strongest associations with clinical health outcomes, including the progression of hypertension and diabetes.^6^ Furthermore, a division, based on whether weight regain exceeded or fell below 20% of the maximum weight loss, proved most efficient among dichotomous measures.^6^ Therefore, our study adopted this definition to investigate the relationship between weight regain and serious health outcomes over 30 years. However, the International Federation for the Surgery of Obesity and Metabolic Disorders (IFSO) recently issued guidelines defining weight regain as gaining 30% or more of the initial weight loss after surgery.^21^ Following this recommendation, we repeated our analysis of mortality and observed results that were largely consistent with the initial findings.

Current understanding of the clinical significance of weight regain following weight loss is still limited.^9^ In this study, most cardiovascular risk factors improved by year 10 compared to baseline in both groups, but the regain group exhibited a worse risk profile with more severe dysregulation of glucose and lipid metabolism and higher systolic blood pressure compared to the maintenance group. Besides weight regain, the groups also displayed differences in energy intake and physical activity during follow-up, which may have contributed to differences in risk factor profiles. While these differences point to a higher risk profile, their relatively small magnitude may explain the similar overall mortality rates between the groups. Deaths attributable to cardiovascular disease and cancer—the two leading causes of mortality in high-income countries—showed no significant difference between those who regained weight and those who maintained their weight loss. Cancer incidence was similar between the two groups, whereas the risk of vascular diseases was significantly higher in the regain group, with the most pronounced increase observed for microvascular disease. This may be related to the less significant improvements in glucose metabolism in the regain group, reflecting the well-established link between hyperglycaemia and microvascular disease.^27^

Multiple factors contributing to weight regain have been identified. These include anatomical modifications, genetic factors, nutritional aspects, hormonal changes, metabolic factors, insufficient physical activity, and psychiatric conditions.^3^^,^^9^^,^^28^ In our study, the difference in BMI between the regain and maintenance groups, from year 4, remained largely consistent over the 20-year follow-up period, suggesting that the factors influencing these trajectories are sustained over time. Individuals who experienced weight regain, on average, consumed more calories and engaged in less physical activity during the follow-up period than those in the maintenance group. This difference emphasizes the importance of continuous focus on dietary habits and physical activity for weight management following weight loss interventions. The SOS-study was designed to examine the long-term outcomes after weight loss and was not intended to compare surgical procedures. However, we observed that although weight regain occurred after all types of surgery, it was least common after gastric bypass, supporting the idea that anatomical changes can influence the likelihood of regaining weight. Long-term studies are inevitably influenced by evolving treatment approaches, and we acknowledge the underrepresentation of current surgical techniques as a limitation of our study. However, when comparing the regain and maintenance groups within individuals who underwent the same surgical procedure, we found similar mortality rates. This suggests that the health risks associated with weight regain are not significantly impacted by the specific method of surgical weight loss.

This study has several limitations. First, weight regain was not a predefined analysis, making the current analysis exploratory in nature and the findings may be the result of chance. Second, our study includes older surgical methods, some of which are no longer in use. However, vertical banded gastroplasty, the most commonly performed procedure in this cohort, has been shown to produce weight loss outcomes comparable to those of sleeve gastrectomy.^29^^,^^30^ Third, the generalizability of our findings may be limited, as the study population was predominantly recruited in Sweden and comprised mostly individuals of Scandinavian ancestry. Additionally, consistent with typical bariatric surgery cohorts, the majority of participants were women. Fourth, the number of participants available for analysis was constrained, partly due to missing body weight data at years one and four. Despite these limitations, the study has several notable strengths, including its prospective data collection, the extended follow-up period with repeated health examinations, and the comprehensive and nearly complete data sourced from national Swedish health registers. In addition, in our analyses, we aimed to isolate the specific effects of weight regain on future mortality and health outcomes. To achieve this, we excluded participants who underwent reoperations involving different surgical techniques or procedures that reinstated normal anatomy, regardless of the reason for the reoperation.

The results of this study should not be interpreted to suggest that weight regain has few negative consequences. While our analyses focused on risk of major chronic diseases and mortality, they did not account for other potential adverse effects of weight regain that also warrant consideration. For example, individuals who regain weight may face more severe obesity-related challenges in their daily lives, encounter greater obesity stigma, suffer a decline in quality of life, or experience an increase in psychiatric disorders. Notably, an early report from a subset of the SOS cohort indicated that weight regain was associated with deteriorations in health-related quality of life.^31^ Further research is also warranted to explore whether conditions like arthrosis^32^ and sleep apnea,^33^ are exacerbated in individuals who regain weight. Nonetheless, the emergence of new, effective anti-obesity medications presents promising opportunities to mitigate weight regain.^34^

In conclusion, partial weight regain after substantial weight loss was associated with a less favourable cardiovascular risk profile and an increased incidence of both microvascular and macrovascular diseases; however, no association with life expectancy was observed.

## Contributors

L.M.S.C., M.P., B.C. and K.S. designed the study. L.M.S.C. and I.A. wrote the first version of the paper with input from M.P., B.C and K.S. J.C.A.-A. and M.P have directly accessed and verified the underlying data reported in the manuscript. M.P. was responsible for the statistical analysis. L.M.S.C., J.C.A.-A., P.J. and K.S. organized register linkage with the Swedish authorities. M.P. and I.A. were responsible for the preparation of tables and figures. All authors contributed to the discussion and interpretation of data, critically reviewed the manuscript and approved the submission of the final version.

## Data sharing statement

Restrictions apply to the general availability of the data because of patient agreements and the nature of the data.

## Declaration of interests

B.C. and C.K. are employed by AstraZeneca and hold stocks in the same company. No other conflict of interest relevant to this study was reported.

## References

[1] Bhaskaran K., Dos-Santos-Silva I., Leon D.A., Douglas I.J., Smeeth L. (2018). Association of BMI with overall and cause-specific mortality: a population-based cohort study of 3.6 million adults in the UK. Lancet Diabetes Endocrinol.

[2] Writing group for the Global BMI Mortality Collaboration, Di Angelantonio E., Bhupathiraju Sh N. (2016). Body-mass index and all-cause mortality: individual-participant-data meta-analysis of 239 prospective studies in four continents. Lancet.

[3] van Baak M.A., Mariman E.C.M. (2019). Mechanisms of weight regain after weight loss - the role of adipose tissue. Nat Rev Endocrinol.

[4] Wilding J.P.H., Batterham R.L., Davies M. (2022). Weight regain and cardiometabolic effects after withdrawal of semaglutide: the STEP 1 trial extension. Diabetes Obes Metab.

[5] Webb V.L., Wadden T.A. (2017). Intensive lifestyle intervention for obesity: principles, practices, and results. Gastroenterology.

[6] King W.C., Hinerman A.S., Belle S.H., Wahed A.S., Courcoulas A.P. (2018). Comparison of the performance of common measures of weight regain after bariatric surgery for association with clinical outcomes. JAMA.

[7] Lauti M., Lemanu D., Zeng I.S.L., Su'a B., Hill A.G., MacCormick A.D. (2017). Definition determines weight regain outcomes after sleeve gastrectomy. Surg Obes Relat Dis.

[8] Voorwinde V., Steenhuis I.H.M., Janssen I.M.C., Monpellier V.M., van Stralen M.M. (2020). Definitions of long-term weight regain and their associations with clinical outcomes. Obes Surg.

[9] El Ansari W., Elhag W. (2021). Weight regain and insufficient weight loss after bariatric surgery: definitions, prevalence, mechanisms, predictors, prevention and management strategies, and knowledge gaps-a scoping review. Obes Surg.

[10] Carlsson L.M.S., Sjöholm K., Jacobson P. (2020). Life expectancy after bariatric surgery in the Swedish obese subjects study. N Engl J Med.

[11] Carlsson L.M., Peltonen M., Ahlin S. (2012). Bariatric surgery and prevention of type 2 diabetes in Swedish obese subjects. N Engl J Med.

[12] Sjöstrom L., Peltonen M., Jacobson P. (2012). Bariatric surgery and long-term cardiovascular events. JAMA.

[13] Sjöstrom L., Peltonen M., Jacobson P. (2014). Association of bariatric surgery with long-term remission of type 2 diabetes and with microvascular and macrovascular complications. JAMA.

[14] Carlsson L.M., Sjöholm K., Karlsson C. (2017). Long-term incidence of microvascular disease after bariatric surgery or usual care in patients with obesity, stratified by baseline glycaemic status: a post-hoc analysis of participants from the Swedish Obese Subjects study. Lancet Diabetes Endocrinol.

[15] Sjöstrom L., Gummesson A., Sjöstrom C.D. (2009). Effects of bariatric surgery on cancer incidence in obese patients in Sweden (Swedish Obese Subjects Study): a prospective, controlled intervention trial. Lancet Oncol.

[16] Aminian A., Wilson R., Al-Kurd A. (2022). Association of bariatric surgery with cancer risk and mortality in adults with obesity. JAMA.

[17] Syn N.L., Cummings D.E., Wang L.Z. (2021). Association of metabolic-bariatric surgery with long-term survival in adults with and without diabetes: a one-stage meta-analysis of matched cohort and prospective controlled studies with 174 772 participants. Lancet.

[18] Brantley P.J., Waldo K., Matthews-Ewald M.R. (2014). Why patients seek bariatric surgery: does insurance coverage matter?. Obes Surg.

[19] Tolvanen L., Svensson A., Hemmingsson E., Christenson A., Lagerros Y.T. (2021). Perceived and preferred social support in patients experiencing weight regain after bariatric surgery-a qualitative study. Obes Surg.

[20] Sjöström L., Larsson B., Backman L. (1992). Swedish obese subjects (SOS). Recruitment for an intervention study and a selected description of the obese state. Int J Obes Relat Metab Disord.

[21] Salminen P., Kow L., Aminian A. (2024). IFSO consensus on definitions and clinical practice guidelines for obesity management-an international Delphi Study. Obes Surg.

[22] Brooke H.L., Talback M., Hornblad J. (2017). The Swedish cause of death register. Eur J Epidemiol.

[23] Ludvigsson J.F., Andersson E., Ekbom A. (2011). External review and validation of the Swedish national inpatient register. BMC Public Health.

[24] Barlow L., Westergren K., Holmberg L., Talback M. (2009). The completeness of the Swedish cancer register: a sample survey for year 1998. Acta Oncol.

[25] Fine J.P., Gray R.J. (1999). A proportional hazards model for the subdistribution of a competing risk. J Am Stat Assoc.

[26] Karmali S., Brar B., Shi X., Sharma A.M., de Gara C., Birch D.W. (2013). Weight recidivism post-bariatric surgery: a systematic review. Obes Surg.

[27] Stratton I.M., Adler A.I., Neil H.A. (2000). Association of glycaemia with macrovascular and microvascular complications of type 2 diabetes (UKPDS 35): prospective observational study. BMJ.

[28] Athanasiadis D.I., Martin A., Kapsampelis P., Monfared S., Stefanidis D. (2021). Factors associated with weight regain post-bariatric surgery: a systematic review. Surg Endosc.

[29] Sjöstrom L. (2013). Review of the key results from the Swedish Obese Subjects (SOS) trial - a prospective controlled intervention study of bariatric surgery. J Intern Med.

[30] Pullman J.S., Plank L.D., Nisbet S., Murphy R., Booth M.W.C. (2023). Seven-year results of a randomized trial comparing banded roux-en-Y gastric bypass to sleeve gastrectomy for type 2 diabetes and weight loss. Obes Surg.

[31] Karlsson J., Taft C., Ryden A., Sjöstrom L., Sullivan M. (2007). Ten-year trends in health-related quality of life after surgical and conventional treatment for severe obesity: the SOS intervention study. Int J Obes.

[32] Reyes C., Leyland K.M., Peat G., Cooper C., Arden N.K., Prieto-Alhambra D. (2016). Association between overweight and obesity and risk of clinically diagnosed knee, hip, and hand osteoarthritis: a population-based cohort study. Arthritis Rheumatol.

[33] Peromaa-Haavisto P., Luostarinen M., Juusela R., Tuomilehto H., Kossi J. (2024). Obstructive sleep apnea: the effect of bariatric surgery after five years-A prospective multicenter trial. Obes Surg.

[34] Elmaleh-Sachs A., Schwartz J.L., Bramante C.T., Nicklas J.M., Gudzune K.A., Jay M. (2023). Obesity management in adults: a review. JAMA.

