# Supplementary material for: Health outcomes and their association with weight regain after substantial weight loss in Sweden: a prospective cohort study
Source: Lancet Reg Health Eur. 2025 Mar 13;52:101261. doi: 10.1016/j.lanepe.2025.101261 (PMC11957514; doi:10.1016/j.lanepe.2025.101261)
Supplement: Supplementary Tables and Figures [file mmc1.docx]

**Supplementary Appendix**

**Health outcomes and their association with weight regain after substantial weight loss in Sweden: a prospective cohort study**

by

Carlsson LMS. et al.

**Table of Contents**

|  | **Page** |
| --- | --- |
| **Figure S1.** Flowchart. | 2 |
| **Figure S2.** Waist circumference. | 3 |
| **Figure S3.** Measures of glucose metabolism. | 4 |
| **Figure S4.** Measures of lipid metabolism. | 5 |
| **Figure S5**. Systolic and diastolic blood pressure. | 6 |
| **Figure S6**. Sensitivity analysis of major adverse cardiovascular events, cancer and microvascular disease. | 7 |
| **Figure S7**. Overall mortality. | 8 |
| **Figure S8**. Sensitivity analysis of survival. | 9 |
| **Figure S9**. Overall mortality after different types of bariatric surgery. | 10 |
| **Figure S10.** Cause-specific mortality. | 11 |
| **Table S1.** International Classification of Diseases and intervention codes. | 12 |
| **Table S2.** Individuals with available data during follow-up | 14 |

**Figure S1. Flowchart.** Study participants in the surgery group who regained 20% or more of their one-year weight loss by the 4-year examination were assigned to the weight regain group, while those who regained less than 20% formed the weight maintenance group.

** Figure S2. Waist circumference.** The comparison between the regain and the maintenance group was adjusted for age and sex.

**Figure S3. Measures of glucose metabolism.** The comparisons between the regain and the maintenance group were adjusted for age and sex.

**Figure S4. Measures of lipid metabolism.** The comparisons between the regain and the maintenance group were adjusted for age and sex.

**Figure S5**. **Systolic and diastolic blood pressure.** The comparisons between the regain and the maintenance group were adjusted for age and sex.

**Figure S6**. **Sensitivity analysis of major adverse cardiovascular events, cancer and microvascular disease.** The regain group includes individuals who regained 30% or more of their one-year post-surgery weight loss by the 4-year examination. Individuals with an event during the first four years were excluded from the respective outcome analysis.

**Figure S7**. **Overall mortality in the regain, maintenance and control group.** After excluding those who underwent bariatric surgery during follow-up, there were 1,365 participants (946 women and 419 men) in the control group, receiving conventional obesity treatment at their primary health care centers. At baseline, the mean age was 49.5 years (SD 6.1), the mean BMI was 39.7 (SD 4.5), and the mean waist-hip ratio was 1.0 (SD 0.1). Serum cholesterol was 5.6 mmol/L (SD 1.1), blood glucose was 4.9 mmol/L (SD 1.8), serum insulin was 17.9 μU/mL (SD 11.0), and HbA1c was 40.6 mmol/mol (SD 10.8). Additionally, 15% (n=208) had type 2 diabetes, 26% (n=354) had impaired glucose tolerance, 66% (n=903) had hypertension, 2% (n=27) had history of cardiovascular disease, 1% (n=14) had cancer before baseline and 19% (n=260) were daily smokers.

**Figure S8**. **Sensitivity analysis of survival.** Shown are the Kaplan-Meier estimate and estimate of survival from an unadjusted Gompertz regression model (fainter lines). The regain group includes individuals who regained 30% or more of their one-year post-surgery weight loss by the 4-year examination.

**Figure S9**. **Overall mortality after different types of bariatric surgery.** Banding (A), Vertical banded gastroplasty (B) and gastric bypass (C).

**Figure S10**. **Cause-specific mortality.** Mortality was classified into cardiovascular, cancer, and other causes.

## **Table S1. International Classification of Diseases and Intervention Codes.** Registry searches were performed using these codes and any sub-classifications thereof.

|  | **ICD-9** | **ICD-10** | **Procedure codes of the National Swedish Board of Health and Welfare** | |
| --- | --- | --- | --- | --- |
| **CARDIOVASCULAR** |  |  |  |  |
| Myocardial infarction  Intracerebral bleeding  Cerebral artery occlusion  Acute but unspecified stroke in terms of bleeding or occlusion  Heart failure | 410  431  433-434  436  428 | I21-I22  I61  I63, I65-I66  I64  I50 |  |  |
| **MICROVASCULAR** |  |  | Classification of operations‡ Ed. 5. 1985 & 6. 1989 (both including also non-surgical procedures) | Classification of surgical procedures (KKÅ) 1997. Temporary list of non-surgical procedures (TÅL) 1997. Swedish Classifications of Health Interventions (KVÅ) 2007^#^ including both surgical (KKÅ) and non-surgical (KMÅ) procedures. |
| Kidney complications | 250D  V42A  791A  584-586  V45B  V56A  V56W | E11.2  E10.2*  E14.2  Z94.0  N08.3  R80  N39.1  N17-N19  Z99.2  Z49 | 6070  6080-6081  9211-9214 | KAS00  KAS10  KAS20  KAB00-KAB01  DR015-DR016  DR020  V9211-V9212  V9507  DR023-DR024  JAK10  TJA20  TJA33  V9213-V9214  V9531-V9532 |
| Eye complications | 250E | E11.3  E10.3*  E14.3  H28.0  H36.0 | 1630-1638 | CKC-CKD |
| Neurological complications | 250F  357E | E11.4  E10.4*  E14.4  G73.0  G99.0  G59.0  G63.2 |  |  |

‡ First edition of Classification of Operations (Swedish: “Klassifikation av Operationer”) was printed by the National Swedish Board of Health and Welfare in 1963.

**#** KVÅ is available only online (http://www.socialstyrelsen.se/statistik-och-data/klassifikationer-och-koder/kva) and is updated annually since 2007. Older code lists were printed by the National Swedish Board of Health and Welfare.

* In the Swedish National Patient Registry and the Cause of Death Registry, complications of some typical type 2 diabetic individuals have erroneously been coded as type 1 diabetes (i.e. with E10# codes), particularly if they have obtained insulin treatment. We have therefore included both E11 (type 2) and E10 (type 1) codes in our searches for microvascular events.

**sTable 2.** The number of individuals with available data at different follow-up time points

|  | **Follow-up year** | | | | | | | |
| --- | --- | --- | --- | --- | --- | --- | --- | --- |
|  | **0** | **1** | **2** | **3** | **4** | **6** | **8** | **10** |
| **BMI**^α^ |  |  |  |  |  |  |  |  |
| Maintenance | 631 | 631 | 620 | 591 | 631 | 536 | 500 | 490 |
| Regain | 715 | 714 | 702 | 677 | 714 | 612 | 591 | 575 |
| **Self-reported energy intake**^β^ |  |  |  |  |  |  |  |  |
| Maintenance | 631 | 605 | 619 | 585 | 572 | 523 | 499 | 490 |
| Regain | 715 | 699 | 693 | 661 | 652 | 624 | 590 | 584 |
| **Leisure-time physical activity**^β^ |  |  |  |  |  |  |  |  |
| Maintenance | 630 | 606 | 619 | 586 | 571 | 523 | 498 | 491 |
| Regain | 713 | 700 | 693 | 661 | 652 | 623 | 590 | 586 |
| **Work-related physical activity**^β^ |  |  |  |  |  |  |  |  |
| Maintenance | 629 | 606 | 619 | 586 | 571 | 523 | 498 | 491 |
| Regain | 714 | 700 | 694 | 661 | 652 | 622 | 590 | 586 |

^α^ Figure 1

^β^ Figure 2
